# Exploring the Applicability of Robot-Assisted UV Disinfection in Radiology

**DOI:** 10.3389/frobt.2020.590306

**Published:** 2021-01-06

**Authors:** Conor McGinn, Robert Scott, Niamh Donnelly, Kim L. Roberts, Marina Bogue, Christine Kiernan, Michael Beckett

**Affiliations:** ^1^School of Engineering, Trinity College Dublin, Dublin, Ireland; ^2^Akara Robotics, Dublin, Ireland; ^3^Department of Microbiology, Trinity College Dublin, Dublin, Ireland; ^4^School of Medicine, Trinity College Dublin, Dublin, Ireland

**Keywords:** UV robot, COVID-19, UVGI, radiology robot, disinfection methods, robotics, HCAI

## Abstract

The importance of infection control procedures in hospital radiology departments has become increasingly apparent in recent months as the impact of COVID-19 has spread across the world. Existing disinfectant procedures that rely on the manual application of chemical-based disinfectants are time consuming, resource intensive and prone to high degrees of human error. Alternative non-touch disinfection methods, such as Ultraviolet Germicidal Irradiation (UVGI), have the potential to overcome many of the limitations of existing approaches while significantly improving workflow and equipment utilization. The aim of this research was to investigate the germicidal effectiveness and the practical feasibility of using a robotic UVGI device for disinfecting surfaces in a radiology setting. We present the design of a robotic UVGI platform that can be deployed alongside human workers and can operate autonomously within cramped rooms, thereby addressing two important requirements necessary for integrating the technology within radiology settings. In one hospital, we conducted experiments in a CT and X-ray room. In a second hospital, we investigated the germicidal performance of the robot when deployed to disinfect a CT room in <15 minutes, a period which is estimated to be 2–4 times faster than current practice for disinfecting rooms after infectious (or potentially infectious) patients. Findings from both test sites show that UVGI successfully inactivated all of measurable microbial load on 22 out of 24 surfaces. On the remaining two surfaces, UVGI reduced the microbial load by 84 and 95%, respectively. The study also exposes some of the challenges of manually disinfecting radiology suites, revealing high concentrations of microbial load in hard-to-reach places. Our findings provide compelling evidence that UVGI can effectively inactivate microbes on commonly touched surfaces in radiology suites, even if they were only exposed to relatively short bursts of irradiation. Despite the short irradiation period, we demonstrated the ability to inactivate microbes with more complex cell structures and requiring higher UV inactivation energies than SARS-CoV-2, thus indicating high likelihood of effectiveness against coronavirus.

## 1. Introduction

Infectious diseases cause significant clinical and economic burden. This has become increasingly apparent in recent months as the toll of coronavirus known as the severe acute respiratory syndrome coronavirus 2 (SARS-CoV-2) has continued to grow. However, the need for improved infection control methods is not exclusively motivated by the COVID-19 outbreak. A large body of literature illustrates the harmful and costly effects of hospital-acquired infections (HAIs). According to Guest et al. (2019), in an average NHS hospital with 510 beds, there may be 3,683 HAIs per year at a cost of 11.9 million, with 126 HAI-associated deaths. The same study estimated that 4.7% of adult hospitalized patients in the NHS would acquire a HAI during their stay, and 1.7% of frontline staff would acquire one annually.

Patients that contract an infectious disease are normally quarantined and subjected to strictly monitored isolation precautions (involving extensive disinfection protocols) in accordance with their condition. Infectious patients that require procedures (such as biopsy, X-ray, etc.) in other locations of the hospital create a significant logistical challenge as additional cleaning is mandated before and after these patients are treated in these rooms. This problem is especially pronounced in Radiology settings where, according to Mossa-Basha et al. (2020), disinfectant cleaning times can take between 30 and 60 minutes after each patient. These delays have had a devastating effect on patient workflow; NHS statistics show that between February and March 2020, the number of people waiting 6 weeks or more for a scan had nearly tripled. It is common practice for much of the disinfectant cleaning to be performed by radiographers (cleaning staff are rarely permitted to disinfect expensive medical equipment). This is problematic as it places additional pressures on the resources within a Radiology department. Disinfectant cleaning is also limited to surfaces only, since the use of aerosolized disinfectant chemicals would be likely to cause damage to any exposed electronic circuitry (PCs, medical equipment, etc.) in the room.

Advances in technology offers potential for improving standards of infection control, namely: (1) increased effectiveness, (2) reduce the time and resources it takes to perform disinfectant cleaning, and (3) reduce the risks incurred by healthcare workers (including cleaning staff) who are required to occupy the facilities. The third point seems especially important in light of findings by Huang et al. (2020), Chen et al. (2020), and others, that COVID-19 has had significant adverse effects on the mental health of front-line health workers.

Ultraviolet germicidal irradiation (UVGI) is a non-touch disinfection method that uses short-wavelength ultraviolet C (UV-C) light to kill or inactivate microorganisms by destroying nucleic acids and disrupting their DNA, leaving them unable to perform vital cellular functions. UVGI has many compelling advantages including the effectiveness against broad-spectrum organisms, lack of harmful residuals, reduced labor and consumable costs, and relative simplicity of operation within a healthcare environment. Despite a large body of scientific evidence demonstrating its efficacy against a wide range of pathogens, including coronavirus, to the best of the authors' knowledge, its effectiveness in radiology setting has not yet been tested. Furthermore, for reasons later outlined, few, if any, of the commercially available UV robot platforms are well suited for this application.

In this paper, we investigate the effectiveness and feasibility of using a UV disinfectant robot for applications in radiology settings. In so doing, we make three significant contributions. First, we propose a design for a UV robot that can actively control its field of UV irradiation, thereby if used in the manner intended, it may be safe for use around humans. Second, using an embodiment of the proposed design, we demonstrate the efficacy of UVGI in inactivating microorganisms from a wide range of commonly touched surfaces in hospital radiology suites. Third, we test the effectiveness of disinfecting a radiology suite comprising a CT scan machine in a period of <15 minutes, a 2-4X reduction on the time it presently takes according to relevant recent literature.

## 2. Limitations of Current Disinfection Methods

The spread of germs, including SARS-CoV-2, can be reduced through improved hygiene practices (i.e., washing hands, social distancing), wearing PPE (i.e., facemasks) and frequent targeted disinfection cleaning. During the COVID-19 outbreak, global shortages of PPE were widely blamed for the high transmission rates among healthcare workers. Other potentially contributing factors, including poor adherence among healthcare workers to hand-washing protocols (Mortell, 2012 observes that this “theory-practice” gap has long existed) and limitations of conventional disinfection cleaning techniques, received relatively less attention.

The issues associated with current disinfection practices are more fundamental, and comprise both operational and scientific factors. Disinfectant cleaning is most commonly undertaken through the application of chlorine-based chemical agents. For best performance, it is normally recommended that surfaces must be first cleaned with a detergent prior to the application of the disinfectant. For a chemical disinfectant to be effective, it must remain wet on a surface for a pre-specified amount of time (normally around 10 minutes). This is difficult to control for in practice, however, as mops/cloths lose their wetness quickly and wetness can be difficult to gauge when wearing latex gloves. Certain germs may also develop resistance to disinfectant chemicals; for example, Edwards et al. (2016) showed that *Clostridium difficile* can develop resistance to household bleach.

Currently, visual inspection is the standard means of assessing the efficacy of cleaning in hospitals. However, since most common germs are <10 microns in size, it is impossible to tell with the naked eye whether a surface has been thoroughly disinfected or not. Sherlock et al. (2009) compared visual inspection against chemical (ATP), microbial methods, and aerobic colony count (ACC). It was found that visual assessment was an inadequate and subjective means of monitoring the cleanliness of hospitals and what appeared clean to the eye was often below acceptable thresholds under other means of analysis.

When disinfectant cleaning is performed, strict infection control protocols must be followed and cleaners are advised to wear gowns, gloves, protective eye-wear, and a mask at all times. In practice, especially in large rooms and those with many surfaces, the activity is time consuming, physically exerting (especially when wearing PPE) and difficult to perform systematically due to factors including the presence of patients, the coming-and-going of physicians, and myriad other things going on at any given time that can cause distractions.

Traditional methods of disinfectant cleaning are limited to surfaces only. Therefore, they can only be considered effective at protecting against fomite transmission. They are ineffective against aerosol and droplet transmission, which is concerning considering the growing evidence that SARS-CoV-2 is primarily transmitted in aerosol form. Santarpia et al. (2020) recently demonstrated the infectious nature of SARS-CoV-2 aerosol, suggesting that airborne transmission of COVID-19 is possible, and that aerosol prevention measures are necessary to effectively reduce the spread of SARS-CoV-2. Chemical disinfectant can be dispersed in the air using misting or vaporizing technologies, such as vaporized hydrogen peroxide (VHP). However, this process is highly time consuming (often takes several hours), resource intensive (vents/doors must be blocked) and logistically challenging as the room must be evacuated during use. Furthermore, there are many parts of the hospital where it is not possible to use chemical misting technologies; examples include public and communal areas that cannot be evacuated (such as hallways, waiting areas, ICU) or in rooms comprising equipment with exposed circuitry such as PCs, CT scanners, etc.

## 3. Prior Work

UVGI technology has been successfully deployed in clinical settings for over a decade. The application of the technology has varied from decontaminating medical devices and PPE (see Kac et al., 2010; Moore et al., 2012), sterilizing ambulances (see Lindsley et al., 2018), air sterilization (see Ethington et al., 2018), to being deployed on mobile platforms (including robots) for the purposes of room disinfection (see Miller et al., 2015).

Several clinical studies have explored the effect of the introduction of UV disinfectant technology on clinical outcomes. In a long-term study spanning several years, Haas et al. (2014) found a correlation between the introduction of UV disinfection robot technology in an acute 643-bed medical center and reduced levels of Hospital Acquired Infections (HAIs). Murphy et al. (2019) report similar reductions in HAIs over a 36 month period after the introduction of a UV disinfectant robot in a bone marrow transplant unit.

Other studies have focused on scientific validation of the technology in the field. One study by Yang et al. (2019) found a substantial reduction in surface bacteria after deploying a mobile UV-C disinfection robot in vacated rooms of patients harboring Methicillin-resistant *Staphylococcus aureus* (MRSA), Vancomycin Resistant *Enterococcus* (VRE) and other nosocomial pathogens. Casini et al. (2019) demonstrated that a pulsed-xenon UV-C device was capable of significantly reducing the amount of microorganisms present on high-touch surfaces in several hospital settings including the Intensive Care Unit, operating theater, and patient rooms.

Research from Memarzadeh et al. (2010), Jinadatha et al. (2015), and Boyce (2016) suggests that UVGI is most effectively deployed in combination with conventional friction-based cleaning methods as the low penetrating power of UV-C limits the effectiveness of the systems in areas that are not first manually cleaned. In a study exploring the efficacy of UVGI systems in an ambulance patient compartment, Lindsley et al. (2018) suggest UVGI as a method of whole-compartment disinfection that would act as a supplement to standard cleaning procedure and allow manual cleaning to be focused on areas most prone to contamination.

Elgujja et al. (2020) reviewed the existing literature on UV surface decontamination in a 2020 publication. Several limitations to existing applications of UV surface decontamination are listed, with the key finding being that shadowed areas remain difficult to sterilize. Other limitations include the inability for UV to remove dust or dirt on surfaces, the need to vacate the room for most UVGI applications and high capital costs. The use of HPV as a disinfection agent has advantages in some areas over UV-C, although the process is time-consuming, cannot be used in an occupied room and HVAC systems must be covered during use. A commonly cited issue with existing UVGI systems is the lack of design against shadowed areas.

Research from van Doremalen et al. (2020) suggests the SARS-CoV-2 virus is detectable on hard surfaces up to 72 hours, and in the air for periods of more than 3 hours. Kampf et al. (2020) demonstrated that other human coronaviruses can persist on some surfaces for up to 9 days. Numerous studies have shown that UVGI is effective against viruses from the coronavirus family. Darnell et al. (2004) successfully inactivated SARS-CoV-1, irradiating liquid samples for 15 minutes at a distance of 3 cm, with a corresponding fluence of 3.6144*J*/*cm*^2^. Eickmann et al. (2020) shows that UV-C irradiation led to the inactivation of three single-strand RNA viruses, including SARS-CoV-1. Walker and Ko (2007) demonstrated 254 nm UV-C inactivation of three viral aerosols: MS2 bacteriophage, adenovirus, and MHV coronavirus. The coronavirus was highly sensitive to UV radiation and was inactivated at a much lower irradiation intensity than the MS2 and the adenovirus. All three pathogens indicated a higher susceptibility to UV inactivation in aerosol form than when suspended in a liquid medium regardless of the size of the virus particle, the type of nucleic acid (DNA or RNA) and the viral structure (naked or enveloped). The finding that UV-C irradiation neutralizes aerosolized pathogens faster than surface-bound pathogens is supported by a study by Kesavan et al. (2014) which found that aerosolized spores were inactivated faster when compared to surface-fixed organism, even when accounting for variance in irradiation intensity observed in the aerosol chamber. More recently, Fischer et al. (2020) found that UV was comparable with hydrogen peroxide vapor (HPV) at inactivating SARS-CoV-2 on solid, non-porous surfaces.

In a review of UVGI technology, Miller et al. (2013) acknowledge that UV irradiation carries some potential health risks. However, the paper states that health issues are rare and typically only occur due to improper maintenance procedures. In this article, the authors make reference to First et al. (2005) who examined eye and skin exposure across a range of settings where UVGI was being used, and showed that doses were well below the recommended level. This corresponds to findings by Lai et al. (2018) that also show applied UV fluences are well below the harmful threshold.

## 4. Methods

### 4.1. Requirements Analysis

To understand the technical and operational requirements for a UV disinfectant solution, we engaged with Radiology departments in two Irish hospitals. We conducted several site visits to both hospitals and interviewed key staff including three consultant radiologists, a radiology services manager, four radiographers, and three infection control nurses. These interactions took place in March 2020, as the country was starting to deal with the toll of COVID-19. The infection control mandate in both hospitals was to thoroughly disinfect all accessible surfaces using a chlorine-based cleaning agent after the room was used by patients that had tested positive for COVID (or were suspected to have COVID). In the first hospital, the majority of the disinfectant procedure was carried out by contract cleaners; however, radiographers were still required to wipe down any medical equipment that was in the room. In the second hospital, a smaller regional hospital, all disinfectant cleaning was performed on-site by the radiographers. In both hospitals, according to the people we spoke with, the minimum time this procedure took was 30 minutes. In the larger hospital, we learned it often took far longer (up to 90 minutes) as room turnaround time was heavily dependent on the scheduling of the contract cleaners, who were in high-demand during the COVID-19 pandemic and often arrived late to the room. We learned that these long cleaning times were having a detrimental effect on patient workflow; in both hospitals, capacity of a CT scanner for COVID patients was 1 per hour, where as pre-COVID it was normal to process 4/5 patients in the same time.

Staff in both hospitals acknowledged that these disinfection periods were unsustainable. However, there was recognition that reducing disinfection times may have a limiting effect on the level of disinfection that was performed. One of the hospitals had considered the use of UV disinfectant technology in the past, and had facilitated a trial of the technology at the hospital. This evaluation process involved the formation of a committee (which included representatives of the Radiology department) which ultimately found that while UVGI technology seemed promising, they anticipated significant operational challenges of integrating currently available solutions into their clinical workflow. Issues identified that were of particular relevance for radiology settings included: (1) the majority of systems on the market were not autonomous and had to be pushed in place, increasing labor requirements, (2) the UVGI platforms were physically large, which made them difficult to maneuver around in smaller or cluttered rooms which are common in radiology settings, (3) they required the room to be evacuated during use, which added to the overall cleaning time since clinical staff were unable to prepare for the next patient until the robot had completed its procedure. They also expressed some high-level concerns regarding the effect that the UV irradiation might have on the equipment in the room.

### 4.2. UV Robot Design

Based on the insights gained during the consultancy phase, a list containing five high-level design requirements was formulated. These requirements were then used to benchmark the applicability of existing UV robot systems for potential use-cases in a Radiology setting (Table 1). It emerged that none of the existing off-the-shelf UV disinfectant robots were well suited for this application, as they were either too large to maneuver in tight spaces (> 55 × 55 cm footprint), required rooms to be fully evacuated during use and/or produced light intensity levels (estimated by Lindblad et al., 2020 to be as high as 1,068 *mJ*/*cm*^2^) which indicated they would rapidly exceed the human occupational safety limits outlined in EU directive 2006/25/EC.

**Table 1:** Benchmarking Violet and several existing UV robots against stated design requirements.

	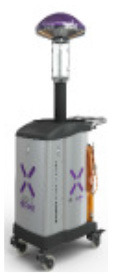	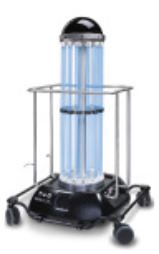	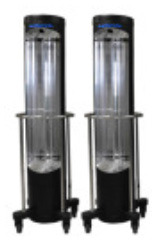	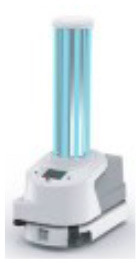	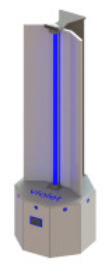
**Robot model**	**Xenex**	**Tru-D**	**Helios**	**UVD**	**Violet**
Form factor (L × W × H)	76 × 51 × 97 cm	NA × NA × 168 cm	59 × 59 × 196 cm	93 × 66 × 171 cm	35 × 35 × 150 cm
Small form factor for operating in tight spaces	X	X	✓	X	✓
Can achieve a greater than 1-log reduction of most germs at distances of 1-2 m from the surface	✓	✓	✓	✓	✓
Can autonomously navigate (i.e., doesn't have to be pushed into place)	X	X	X	✓	✓
Cleaning staff can be deployed in the room at same time as robot	X	X	X	X	✓
UV lamp not too powerful to cause harm if exposed to skin/eyes for short period	X	X	X	X	✓

**Figure 1:**
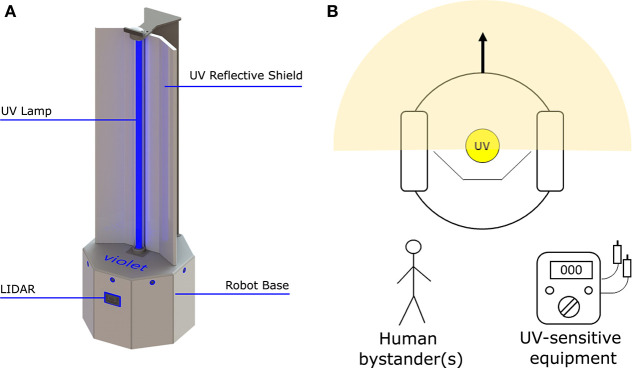
The *Violet* robot platform. **(A)** Labeled image illustrating some of the robot's key features. **(B)** Plan view illustration of how *Violet* uses a physical barrier to shield bystanders and/or equipment from irradiation.

A prototype disinfectant robot was developed to overcome these limitations; our goal was to produce a system that could perform autonomously, was capable (at least potentially) of working safely alongside human cleaners, and that it possessed a small form factor which made it suitable for use in constrained spaces. Our design concept, which we subsequently named *Violet*, is shown in Figure 1. *Violet* comprises a differently driven robotic base equipped with a Hokoyu URG lidar and Intel Realsense D400 RGBD camera for autonomous navigation. It has one (or more as needed) vertically mounted UV lamps enclosed by a reflective shield. This shield serves two important purposes: (1) to reflect UV radiation emitted behind the robot, thus helping to amplify the total UV output (as shown by Miller et al., 2013 and several earlier studies), and (2) to control the parts of the room that are irradiated.

The *Violet* prototype was constructed using a Turtlebot 2 mobile base which mounted a vertical mercury vapor UV lamp (wavelength 254 nm). The lamp was powered through a DC-AC inverter which was connected with a 10Ahr Lithium Polymer battery pack. The robot and UV lamp were controlled using an on-board Hystou mini-pc (Intel i7 processor, 8 GB RAM). A wide angle logitech webcam was located at the front of the robot. The reflective shield was implemented using a folded piece of 1 mm Aluminum sheet metal. Fixtures for mounting the lamp, reflector and sensors were custom fabricated through a combination of laser cutting and 3D printing. The robot could be controlled manually using a Logitech handheld joystick, or autonomously using the Turtlebot's navigation stack which was openly available within the Robot Operating System (ROS) repository. Prior to deployment, the robot was tested in an anechoic chamber to ensure that it didn't produce any RF interference that would cause problems to hospital equipment.

### 4.3. Designing for Occupational Health and Safety

**Figure 2:**
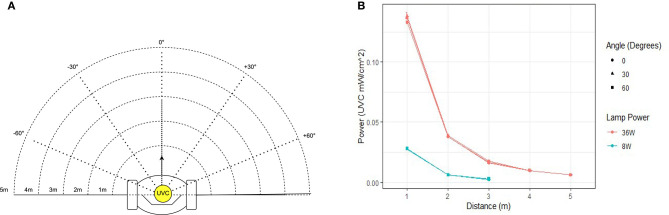
Measured UV-C field in the region around the robot. **(A)** Measurements were taken at distances of 1–5 m at intervals of 30. **(B)** Graph illustrating the UV-C output of the 8 and 36 W lamp used in this study.

The UV-C output of an 8W (model: Philips TUV 8W FAM/10X25BOX) and 36W (Model: Philips TUV 36W SLV) lamp were measured empirically in a series of lab tests. Using a UV-C light intensity meter (Model: General UV254SD), the intensity of UV light was measured over distances of 1–5 m. To ensure that the light irradiated uniformly from the lamp, measurements were taken at 30 degree increments over a span of 120 degrees. Results from this experiment are plotted in Figure 2 and tabulated in Table 2. The power intensity dropped significantly over distance for both 36 and 8 W bulbs. Based on its improved performance over distance, the 36 W lamp was selected for use on the *Violet* prototype.

**Table 2:** Instantaneous UV-C energy field around the robot for an 8 W (model: Philips TUV 8 W FAM/10X25BOX) and 36 W lamps (model: Philips TUV 36 W SLV).

		**1 m [mW/cm**^****2****^**]**		**2 m [mW/cm**^****2****^**]**		**3 m [mW/cm**^****2****^**]**		**4 m [mW/cm**^****2****^**]**		**5 m [mW/cm**^****2****^**]**	
**Angle**	**Power (W)**	**μ**	**σ**	**μ**	**σ**	**μ**	**σ**	**μ**	**σ**	**μ**	**σ**
−60°	8	0.027	0.001	0.006	0.000	0.003	0.000	N/A	N/A	N/A	N/A
	36	0.132	0.000	0.038	0.000	0.017	0.001	0.010	0.000	0.006	0.000
−30°	8	0.027	0.000	0.006	0.000	0.003	0.000	N/A	N/A	N/A	N/A
	36	0.136	0.001	0.038	0.001	0.017	0.000	0.009	0.000	0.006	0.000
0°	8	0.028	0.000	0.006	0.000	0.002	0.000	N/A	N/A	N/A	N/A
	36	0.137	0.001	0.038	0.000	0.016	0.000	0.010	0.000	0.006	0.000
30°	8	0.028	0.000	0.006	0.000	0.002	0.001	N/A	N/A	N/A	N/A
	36	0.141	0.001	0.039	0.000	0.018	0.000	0.010	0.000	0.006	0.000
60°	8	0.029	0.000	0.006	0.000	0.003	0.000	N/A	N/A	N/A	N/A
	36	0.134	0.001	0.038	0.000	0.016	0.000	0.009	0.000	0.006	0.000

*The highest detected output was 7.46 mW/cm^2^, measured at a distance of approximately 5 mm from the 36 W lamp*.

The UV light intensity meter was also used to test the effectiveness of disposable gloves, a range of clothes fabrics and standard plastic PPE googles to shield UV rays. Our tests found that even at distances of just a few centimeters, the UV meter was unable to register a reading for any of the materials tested.

The legal daily exposure limit (over an 8 hour period) of unprotected skin and eyes, as per the EU Directive 2006/25/EC, is an effective radiant exposure value, *H*_*eff*_ of 30*J*/*m*^2^. For a UV-C light source, this can be calculated by the following equation:

(1) $$\begin{array}{l} H_{eff}=\Sigma_{\lambda =180nm}^{\lambda =400nm}E_{\lambda }\cdot S\left(\lambda \right)\cdot \Delta \lambda \cdot \Delta t \end{array}$$

Where *E*_λ_ = spectral power density (*Wm*^−2^*nm*^−1^), *S*(λ) = spectral weighting accounting for wavelength dependence of health effects of UV radiation on skin and eyes (dimensionless), Δλ = bandwidth of measurement intervals (*nm*), and Δ*t* = duration of exposure (*s*). Using the formula provided in Equation (1), it was estimated that if *Violet* was equipped with one Philips TUV226 36W SLV lamp, reaching this exposure limit would take 44 seconds at a distance of 1m, 2 minutes 37 seconds at 2 m, and 5 minutes 57 seconds at 3 m.

### 4.4. Sample/Data Collection and Analysis

The germicidal effectiveness of the *Violet* robot was examined at two hospital sites. Surfaces to be sampled were divided into eight 1 × 1 cm squares. Swabs, moistened with phosphate buffered saline (PBS), were used to sample 4 alternate squares before UV irradiation. Sample swabs were transferred to 1 ml PBS and stored on ice. After irradiation, surfaces were then re-sampled (using the four remaining squares at each sample location) using the same approach followed in the pre-treatment phase. Collected samples were diluted 1:10 in PBS and 100 μl was plated onto nutrient agar with four technical replicates and grown statically for 48 h at 37°C. Bacterial load was reported as colony forming units per square centimeter (*cfu*/*cm*^2^) after correction for dilution and surface area.

## 5. Results

A series of tests were undertaken within the Radiology departments of two Irish hospitals; a 500+ bed general hospital in Dublin (hospital 1) and a 250+ bed regional hospital (hospital 2). The first tests, performed at hospital 1, were designed with a focus on validating the germicidal efficacy of the *Violet* system. The second tests, which were performed in hospital 2, had a greater focus on validating the operational feasibility of using a UV robot to autonomously disinfect a room in a real-world, clinical radiology setting.

### 5.1. Experiment 1

**Figure 3:**
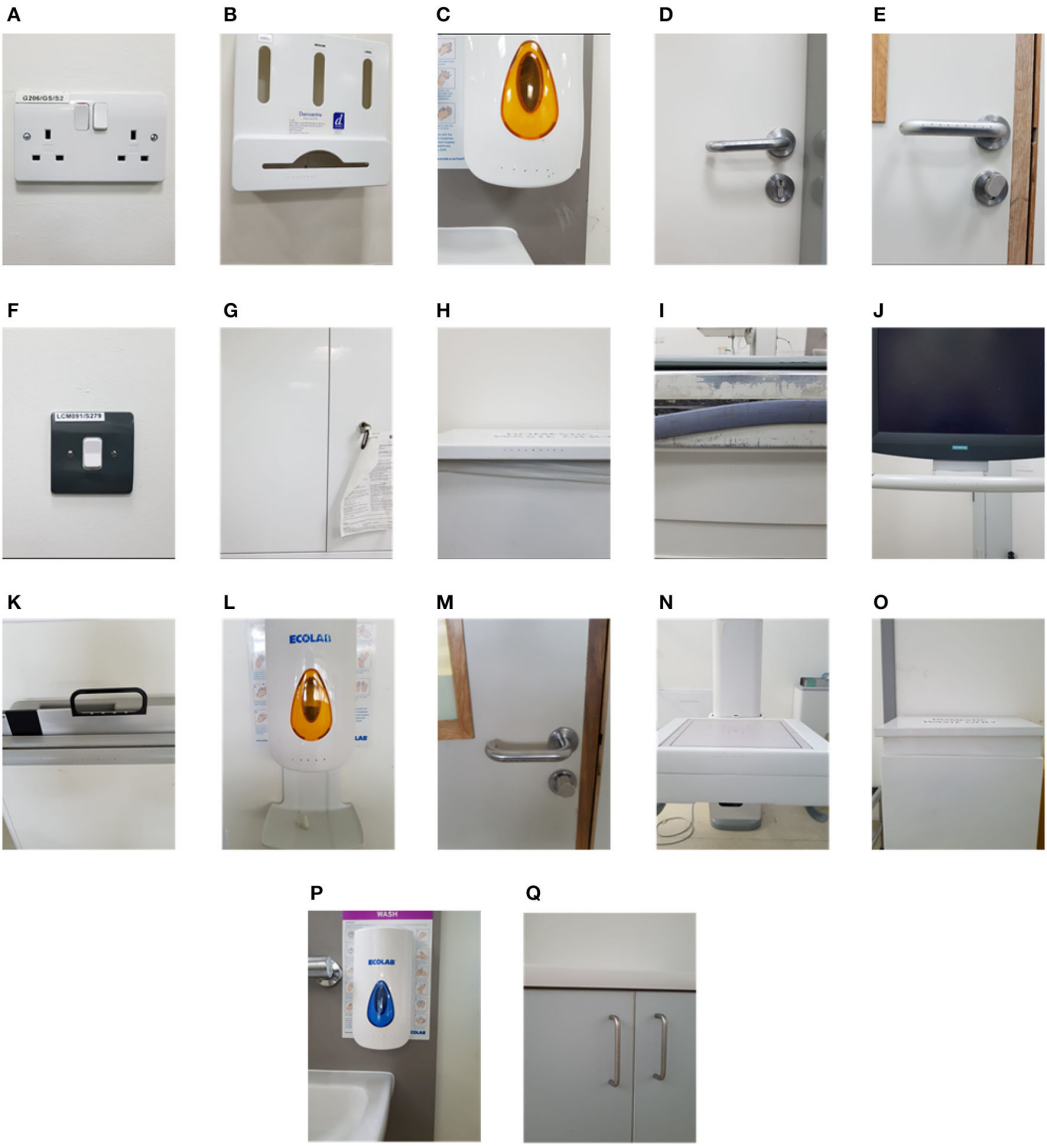
Commonly touched surfaces chosen for swab testing in hospital 1: **(A–J)** CT scan room; **(K–Q)** interventional suite.

**Figure 4:**
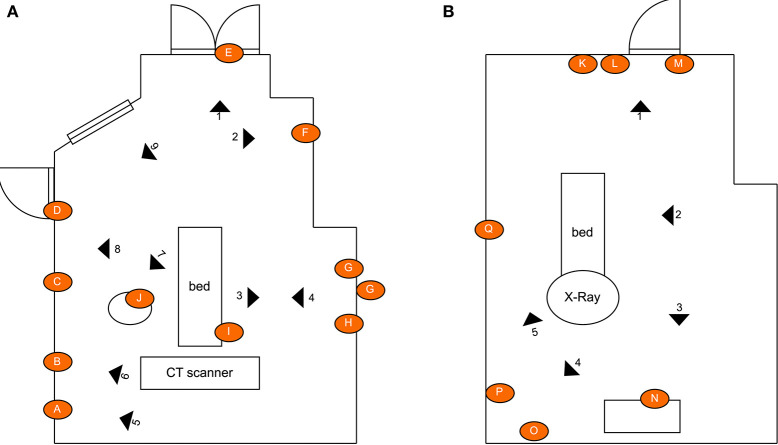
Schematic illustrating the layout of the rooms, the location of the swab points in each room, and the approximate location of the robot during the defined irradiation periods: **(A)** CT scan room; **(B)** interventional suite. The swept area of the UV lamp is illustrated for location 1 to provide an indication of the volume of space covered. [Drawings not to exact scale].

The first experiment involved irradiating two radiology suites at hospital 1; a CT-scan room and X-ray room. Both rooms were estimated to be 34–40*m*^2^ in area. The disinfection treatment involved navigating the robot to several locations (10 in CT scan room, 7 in the interventional suite) to irradiate nearby surfaces. To ensure repeatability and controllability of the experiment, the robot was manually controlled by a human operator. The robot stopped for a period of 3 minutes at each location. The stopping locations were chosen in advance, and selected as such that they irradiated many of the frequently touched surfaces in the room. Surfaces that were selected for swab testing had a high probability of human skin contact and were distributed through the room. Surfaces of medical equipment (including the CT scanner tunnel) were not subjected to high doses of irradiation. This was introduced as precautionary measure in case that a high dosage of UVC light might have a degrading effect on medical equipment. Photographs of the surfaces sampled from the CT room and X-ray room of hospital 1 are given in Figure 3. A schematic illustrating the positioning of the swab points in each room, as well as the approximate locations of the robot during the experiment are presented in Figure 4.

**Figure 5:**
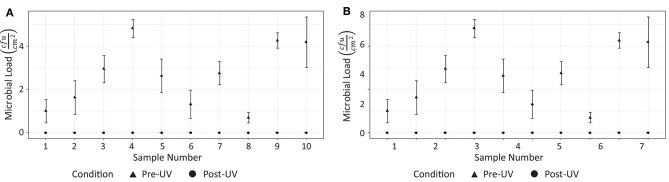
Effectiveness of UV irradiation at eliminating on bacterial load on surfaces in hospital 1: **(A)** CT suite, **(B)** X-ray suite.

Analysis of the swab samples revealed that, of the microbes present before the irradiation, a mix of both Gram positive and Gram negative bacteria was detected. Results from the swab testing indicates that UV irradiation successfully eliminated all of the measurable bacterial load on each of the different surfaces tested. The presence of *Staphylococcus aureus*, was detected on the door handle of the X-ray room (Figure 3M) prior to, but not after, UV irradiation. These results are shown in Figure 5.

### 5.2. Experiment 2

**Figure 6:**
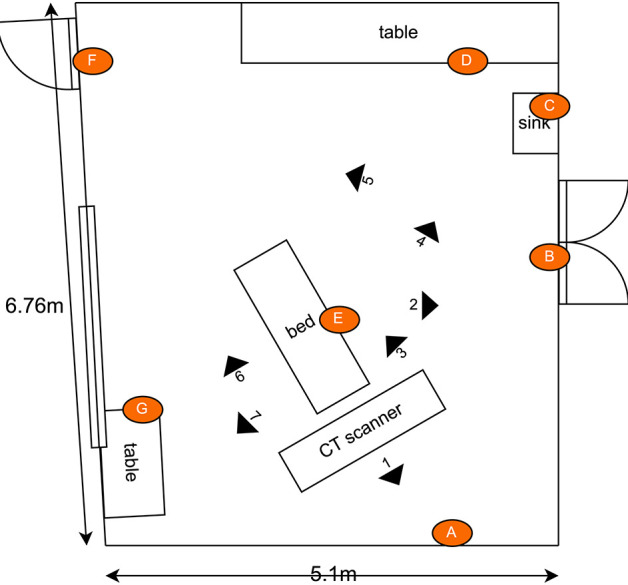
Schematic illustrating the layout of the CT scan room used for the second hospital test. Indicated in the drawing are the locations of the swab points, and the approximate location of the robot during the defined irradiation periods. [Drawings not to exact scale].

**Figure 7:**
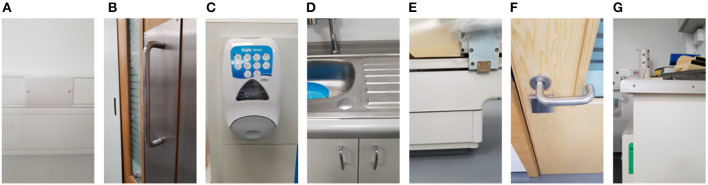
Commonly touched surfaces **(A–G)** chosen for swab testing in CT scan room of hospital 2.

The second experiment investigated the germicidal effectiveness of a rapid (<15 minutes) CT room disinfection using the *Violet* robot. This experiment involved navigating the robot throughout the CT room, stopping at seven locations to irradiate nearby surfaces. The trajectory of the robot, including its irradiation locations, were chosen in advance to maximize the total surface area exposed to UV irradiation. The robot stopped for a period of 2 minutes per location. The irradiation locations and the swab points were chosen independently of the route followed by the robot. To ensure repeatability and controllability of the experiment, the robot was manually controlled by a human operator. A schematic of the room, which shows the positioning of the swab points, as well as the approximate irradiation locations of the robot during the experiment are presented in Figure 6. Photos of the swab locations are given in Figure 7. The feasibility of autonomously performing the procedure was separately validated; a video showing *Violet* performing a similar route to the one described in the paper is given in the Supplementary Material.

**Figure 8:**
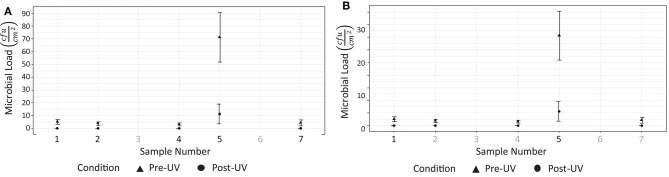
Results from testing at hospital 2. **(A)** Reduction of measurable bacterial load on surfaces after 15 disinfection period (testing day 1). **(B)** Reduction of measurable microbial load on surfaces after 15 disinfection period (testing day 2).

Tests were performed on two occasions, 1 week apart. These results are shown in Figure 8. In the first test, swabs taken from two locations (Figures 7C,F) did not reveal the presence of any microbial load prior to the UV irradiation. On the second day, swabs from five locations (Figures 7B–D,F,G) did not reveal the presence of any bacteria prior to the UV irradiation. At the remaining locations, the UV irradiation was shown to be highly effective at eliminating microbial load. At one location, in a crevice on the bed of the scanner (Figure 7E), an especially high concentration of bacterial load was measured on both days; this included both *S. aureus* and *Staphylococcus epidermidis*. The UV irradiation eliminated 84% of the bacterial load at this location on testing day 1, and 95% of the bacterial load on testing day 2.

## 6. Discussion

The results from our analysis indicate that robot-assisted UVGI can be an effective tool of reducing the presence of microorganisms in radiology departments. Our findings are likely the first to show effectiveness of UVGI in a radiology context, however they add to a growing body of research that demonstrates effectiveness of the technology within clinical settings.

Pre-UVGI swab testing revealed the presence of microbial load at (24/31) locations across three radiology suites. After UVGI treatment, microbial load was only detected at (2/24) locations. The location where the greatest concentration of microbial load was observed was on the patient bed in hospital 2, at a location adjacent to a crevice at the bed's lifting mechanism (Figure 7E). Both *S. auris* and *S. epidermidis* were also observed at this location. The high microbial load suggests that the site may have been inaccessible to human fingers during normal cleaning procedures. Although the UVGI didn't fully remove the microbial load at this location, it was successful in reducing it by >85% in both instances. The most plausible reason why the UVGI did not eliminate all bacteria at this location was due to shadowing in the vicinity of the crevice where the swab samples were taken. This limitation could be addressed through better placement of the UV lamp and/or a longer exposure time.

It was not possible to directly measure the effectiveness of *Violet* against coronavirus, since its presence in uncontrolled real-world settings is hard to quantify. However, we can estimate the likely performance by comparing the UV-C output of the robot with inactivation energy to kill the virus and based on its performance at inactivating microbes with more complex cell structures. At a distance of 2 m, using the data given in Figure 2, we would expect *Violet* to generate a UV-C dose of approximately 4.2 *mJ*/*cm*^2^ over a 2 minutes period. This is higher than the inactivation energy to achieve a log reduction of SARS-CoV-2, which was estimated by Heßling et al. (2020) to be 3.7 *mJ*/*cm*^2^). Furthermore, we observed that the system was successful at inactivating *S. auris*, which, according to Kowalski (2010), has a UV inactivation energy for log reduction of 6.06 *mJ*/*cm*^2^.

UVGI systems are widely used for air purification applications, so it is likely that a system like *Violet* will be effective at inactivate microbes in the air as well as on surfaces. However, the extent to which the methods used in this study may lead to measurable reductions of microbial load in air remains untested. As the distribution of microbes in the air is likely to fluctuate with changing air currents, measuring this would necessitate a different study design and was therefore beyond the scope of this paper. The authors think this would be an interesting piece of research for future work.

Our research provides evidence that relatively small doses of UV-C irradiation, suitably administered at short distances from target surfaces, can achieve significant reductions in microbial load. However, since relatively few studies have quantified the performance of manual disinfection methods, further work is needed in order to benchmark this performance with current disinfection standards. It is noted that in the preparation for this study, the authors performed a similar swab analysis to the one described in this work before and after a manual deep-clean of CT scan treatment room. We detected microbial load at four of the seven locations sampled. At these sample sites, the disinfectant cleaning only reduced the concentration by 10–30% at each location, indicating significantly worse performance than observed using UVGI in our experiments. It is not possible to draw conclusions from a single test, however this preliminary finding motivates follow-on work which should compare, where possible, the performance of UVGI relative to a benchmark of currently deployed cleaning methods.

It has been identified that a limitation of UVGI is that only surfaces that are in direct view of the light are irradiated, thus shadowing can prevent some surfaces being disinfected. Using a mobile robot base helped to mitigate this problem, since surfaces obscured when the robot was in one location, may not have been when the robot was in an adjacent position. However, there are certain features, such as the underside of door handles, where moving is not likely to affect things significantly. It may be possible to overcome this limitation with careful installation of reflective surfaces that could reflect UV light onto surfaces that may be otherwise obscured from the exposed lamp; the effectiveness of this approach merits further investigation. Similarly, it is conceivable that certain pieces of equipment, such as medical devices, may still necessitate manual cleaning regardless of whether they are irradiated by UV-C. Since these areas represent relatively small sections within the room, this should only demand to a small amount of human time. For example, we estimate in the radiology setting explored in this study, it would take approximately 1 minutes for a person to wipe down medical equipment and parts of the room that the light from the robot did not irradiate. This does, however, reinforce the need for coordination between the robot and the person tasked with cleaning the remaining parts of the room.

Through the addition of physical shielding on the robot, it was possible to control parts of the room that were irradiated with UV-C light. Since background radiation was found to be negligible, if implemented alongside suitable safety protocols, it's conceivable that a UVGI robotic system with a similar design may be deployed safely alongside staff, patients, or other hospital users. This feature would have an immediate advantage of increasing capacity within a radiology context, since it would enable staff to perform critical room preparation tasks at the same time that robot was disinfecting, thus shortening the turn-around time for the room. This capability may make the technology more accessible for use in settings like ICU where the incidence of HAIs is typically high and it is not normally possible to evacuate ventilated patients from the room to perform disinfectant cleaning. This design may also be advantageous for disinfecting public parts of the hospital (i.e., waiting areas, cafeterias, receptions, hallways) as well in non-clinical settings (i.e., trains, retail, airports) where frequent disinfection is needed but where it may not be possible to evacuate the space on a high-frequency basis.

The findings from this study raise several new research questions worth separate investigation. First, it would be interesting to know the time effects of UVGI treatment, namely if and for how long any germicidal effects of UVGI may persist after application. Second, with several different UV disinfectant robots on the market, and many with distinct designs, it would be of great interest to both the infection control community, as well as to hospital purchasing departments, to benchmark the germicidal performance of different UV disinfection robots from trials conducted in the field. It is noted that this paper is not the first to call for greater benchmarking; Masse et al. (2018) made the same observation and have already investigated the germicidal performance of two existing solutions (not capable of autonomous navigation) that are comparable with the robot in this study. Third, the findings in this work motivate a follow on clinical trial, whereby the germicidal performance of the UVGI system is measured directly against current disinfection procedures within radiology. Finally, while this study focused on a radiology department use-case, we have added to the body of scientific knowledge showing that the technology has the potential to be a powerful tool for limiting the spread of harmful pathogens, and may have a wide range of applications beyond radiography. As yet, few studies have yet investigated the effectiveness of UV disinfectant in non-clinical settings. The authors propose that testing in areas including on public transport, in nursing homes and long-term care, and in public buildings/schools/colleges would be interesting work.

## 7. Conclusions

Effectively preventing the spread of infectious diseases, such as COVID-19, requires the utilization of air/surface disinfectant practices as well as behavioral changes such as social distancing and cocooning. Currently, only chemical-based disinfectant practices are being widely used and recommended by advisory groups such as the CDC and EPA. Alternative non-touch methods of disinfectant, such as ultraviolet germicidal irradiation, have the potential to overcome many of the practical limitations of chemical-based approaches, and may be automated for use in a much wider variety of settings where rigorous disinfectant protocols were previously not feasible, such as on public transport, nursing homes and in schools/universities. Our findings suggest that UVGI can effectively inactivate germs on commonly touched surfaces in radiology suites, even if the surfaces were not cleaned in advance and only exposed to relatively short bursts of irradiation. These results add to the growing body of scientific literature that supports the efficacy of UVGI in clinical settings. Our study also demonstrates the feasibility of using a bespoke robotic-UVGI system to reduce the time taken to disinfect rooms; in this study, we provide evidence that a comprehensive disinfectant procedure, that can operate in both the air and on surfaces, could feasibly be undertaken in 15 minutes or less. This new disinfectant approach is estimated to be between two and four times faster than currently-used chemical approaches. If such a system were implemented, it could both significantly improve workflow and machine utilization, and reduce exposure of front-line healthcare workers to infectious pathogens.

## Data Availability Statement

The raw data supporting the conclusions of this article will be made available by the authors, without undue reservation.

## Author Contributions

CM and MBe conceived of the presented idea. CM and ND developed robot used in the testing. CM, ND, MBe, and CK performed interviews and site visits to hospital locations. KR advised and assisted on the theory and analytical methods. CM and MBe conducted all microbial testing and analysis. All authors (including MBe and MBo) discussed the results and contributed to the final manuscript.

## Conflict of Interest

Two of the authors have involvement with a robotics company that are active in the development of a robotic disinfectant system. All work relating to the collection and analysis of data was performed by an author that is not involved in this company.
